# Case Report: COVID-19 Infection With Gastrointestinal Symptoms and Mood Disorder: Criticalities in Differential Diagnosis, Therapy and Management of Complications

**DOI:** 10.3389/fpsyt.2021.568553

**Published:** 2021-12-10

**Authors:** Giuseppina Borriello, Lisa Lavatelli, Francesca Ruzzi, Adelaide Panariello, Mauro Emilio Percudani

**Affiliations:** Department of Mental Health and Addiction Services, Niguarda Hospital, Milan, Italy

**Keywords:** gastrointestinal, liver injury, mood disorder, COVID pandemic, eating disorder

## Abstract

During this pandemic Italy was deeply hit by the burden of the COVID-19. Current studies reveal that respiratory symptoms of COVID-19 represent the most common manifestations at presentation. The incidence of less common gastrointestinal symptoms varies significantly among different study populations. Liver injury is also described at different degree. We describe the case of a 20-year-old woman confirmed as SARS-CoV-2 positive by nasopharyngeal swab-PCR test, admitted to the COVID-only—Psychiatric Ward, set up in Niguarda Hospital in Milan on March 2020, for a depressive episode characterized by depressed mood and anorexia. In comorbidity we report a previous avoidant/restrictive food intake disorder present since childhood and a Border Personality Disorder according to the DSM V. On the admission to the ward we administered the Hamilton Depression Rating Scale with a total score of 29 suggesting severe depression. During the hospitalization she developed a clinical picture with increasing vomiting and diarrhea, nausea, abdominal pain along with fever and no respiratory symptoms. She also showed abnormalities in liver function indices. At the same time she showed clinophilia and persistent food avoidance that, initially, led to attribute all the symptoms to her psychiatric disorders. We prescribed the already ongoing therapy with lithium carbonate and SSRI. On the second day of hospitalization, along with the worsening of the gastrointestinal symptoms, we started therapy with hydroxychloroquine with a no significant remission of nausea and vomiting but with a further increase in liver function indices suggesting liver damage. This led us to suspend the treatment with hydroxychloroquine for the suspect of a drug induced injury. The depressive symptoms improved rapidly as opposed to the patient's overall condition. The gastrointestinal symptoms resolved with the evidence of the recovery from infection. In this report we underline the importance of investigating the physical symptoms in a patient with a history of mental disorder especially during an undergoing pandemic. During this pandemic, specialists from various fields were called upon to support teams working with COVID patients and to acquire new skills out of necessity, fostering a multidisciplinary approach and cooperation.

## Introduction

On March 11th 2020 WHO defined COVID-19 as a pandemic. The outbreak began in Wuhan in China in December 2019 and rapidly spread around the world. Italy has been deeply hit by the COVID-19 epidemic, especially in the Northern regions. In Italy 204,000 cases and 27,682 deaths were registered until April 30th. In Lombardy alone, as of this writing, there are 75,732 confirmed cases, with a total of 13,772 deaths^1^.

Ongoing studies have shown that respiratory symptoms of COVID-19 such as fever, dry cough up to dyspnea represent the most common manifestations of the disease, and that are strongly indicative of a transmission of the infection by droplets and by contact^2^. The incidence of gastrointestinal symptoms like diarrhea, nausea, vomiting, and abdominal discomfort is less common and varies significantly between different study populations, along with an early and mild onset frequently followed by the appearance of typical respiratory symptoms (1). Anosmia has also been reported (2).

SARS-CoV-2 attacks the respiratory system as a preferential site, specifically the pulmonary alveoli by binding to ACE2 receptor (3). It showed tropism for various organs including above all heart, kidneys, brain and the gastrointestinal system, especially liver and pancreas (4). This picture is most likely attributable to the ubiquitous distribution of angiotensin-converting enzyme 2 (ACE2). Hepatic involvement is not a major manifestation of COVID-19, it is not yet clear what impact this has on the prognosis of these patients (5).

During COVID-19 outbreak, psychiatric services in Lombardy have been working to guarantee continued services at both a residential and community level, guaranteeing hospitalization for acute cases infected by SARS-CoV-2 (6). The Niguarda Hospital in Milan, has dedicated psychiatric ward for COVID-19 positive patients with acute psychiatric disorders. A local protocol for treatment of psychiatric patients positive for COVID-19 was implemented. Infectious Diseases Unit carried out an initial assessment, providing therapeutic and diagnostic indications, and daily follow-up consultations with 24 h availability. Intensivists visited patients in hospital to evaluate eligibility for therapeutic upgrade; immediately reporting clear evidence of interstitial pneumonia with respiratory distress defined as breath rate >30 acts/minute, SpO2 <93–92%, or when the PaO2/FiO2 ratio of <300 mmHg on blood gas analysis. Hospitalization in the intensive care-unit (ICU) was provided for patients with severe respiratory distress or additional severe COVID-19 symptoms. From March 9th to April 30th 2020, 24 COVID-19 positive patients with acute psychiatric disorders were hospitalized (14 M, 10 F; mean age 41 years; 6 Psychoses, 3 Bipolar disorders, 7 Depressive disorder, 4 Personality disorders; 2 Cognitive disorder; 2 Intellectual disability). None of these had relevant gastrointestinal or hepatic diseases in previous medical history.

Among our patients, a 20-year-old woman reported a prevalent gastrointestinal symptomatology and an important increase in liver function indices in the absence of involvement of the respiratory system. The clinical case we will examine highlights complex problems of differential diagnosis with the main psychiatric disease, critical issues in therapy and in the management of complications that occurred during treatment.

## Case Presentation

The 20-year-old female patient accessed our ward for a picture compatible with recurrent depressive episode in personality disorder. In the days preceding the hospitalization, the patient had visited the general Emergency Department twice reporting fever (max body surface temperature 38°C). Upon entry to the ward, a nasopharyngeal swab test was performed for COVID-19 which tested positive. Anamnesis reports a history of a food intake disorder (restrictive behaviors) (7) present since childhood and a comorbid diagnosis of Border Personality Disorder (8). The patient is known for prior hospitalizations in our ward for depressive symptoms, self-injurious behaviors, hypo/anorexia.

On admission, a psychopathological picture was highlighted, characterized by a marked deflection of mood tone, asthenia, hypo/anorexia, self-injurious ideation, insomnia, overall psychomotor slowing. In the days preceding the hospitalization she had been practicing self-cutting on the left forearm which was being medicated.

In order to complete the psychopathological diagnostic assessment, the Hamilton Depression Rating Scale (9) was administered with a score of 29 suggesting severe depression.

Upon admission to ward, parameters were measured: the patient was paucisymptomatic, with febricula (up to 37.6 °C), oxygen saturation (Sp02) of 100%. Laboratory parameters, in particular complete blood count, liver, kidney and thyroid function indices, coagulation, lipidic and glucidic profile were within normal ranges, with the exception of a modest increase in PCR (3.9) and a mild hypoalbuminemia (3.35 as compared to normal range of 4.02–4.76).

During the hospital stay, as per hospital protocol, vital signs were measured daily, 3 times a day (Blood Pressure, Heart Rate, Respiratory Rate, Sp02, Body Temperature) and blood chemistry tests were carried out every 72 h to monitor SARS-CoV-2 infection, especially blood count with formula, AST, ALT, total and esterified bilirubin, alkaline phosphatase, LDH, electrolytes, creatinine, urea, urate, Pro-BNP, Pro calcitonin, fibrinogen, PT, PTT, D-dimer, glycaemia, CRP, SPE (Table 1). To complete the diagnostic framework, a high-resolution chest CT scan was also performed which did not show characteristic findings for COVID-19.

**Table 1:** Trend of the main health parameters (body temperature, oxygen saturation, heart rate, body weight).

**Nasopharingeal swab-PCR test**	**Body temperature**	**Oxygen saturation**	**Heart rate**	**Body weight**
Positive (16/03)	37.6	99	102	65
Weak positive (02/04)	37	100	100	64.2
Negative (15/04)	36.7	99	70	63.1
Negative (18/04)	37.1	98	79	62.8
Negative (20/04)	36.8	100	56	62.5

Two days after admission, the patient began to show a symptomatology of nausea, vomiting and loss of appetite; the latter was reported also during the previous hospitalizations. She also had bouts of diarrhea. In the last 2 years, the patient had 5 ordinary hospitalizations in our ward, during which an established relational pattern emerged, characterized by a regressive behavior with restricted eating behaviors and self-induced vomiting together with depressive symptoms.

**Figure 1:**
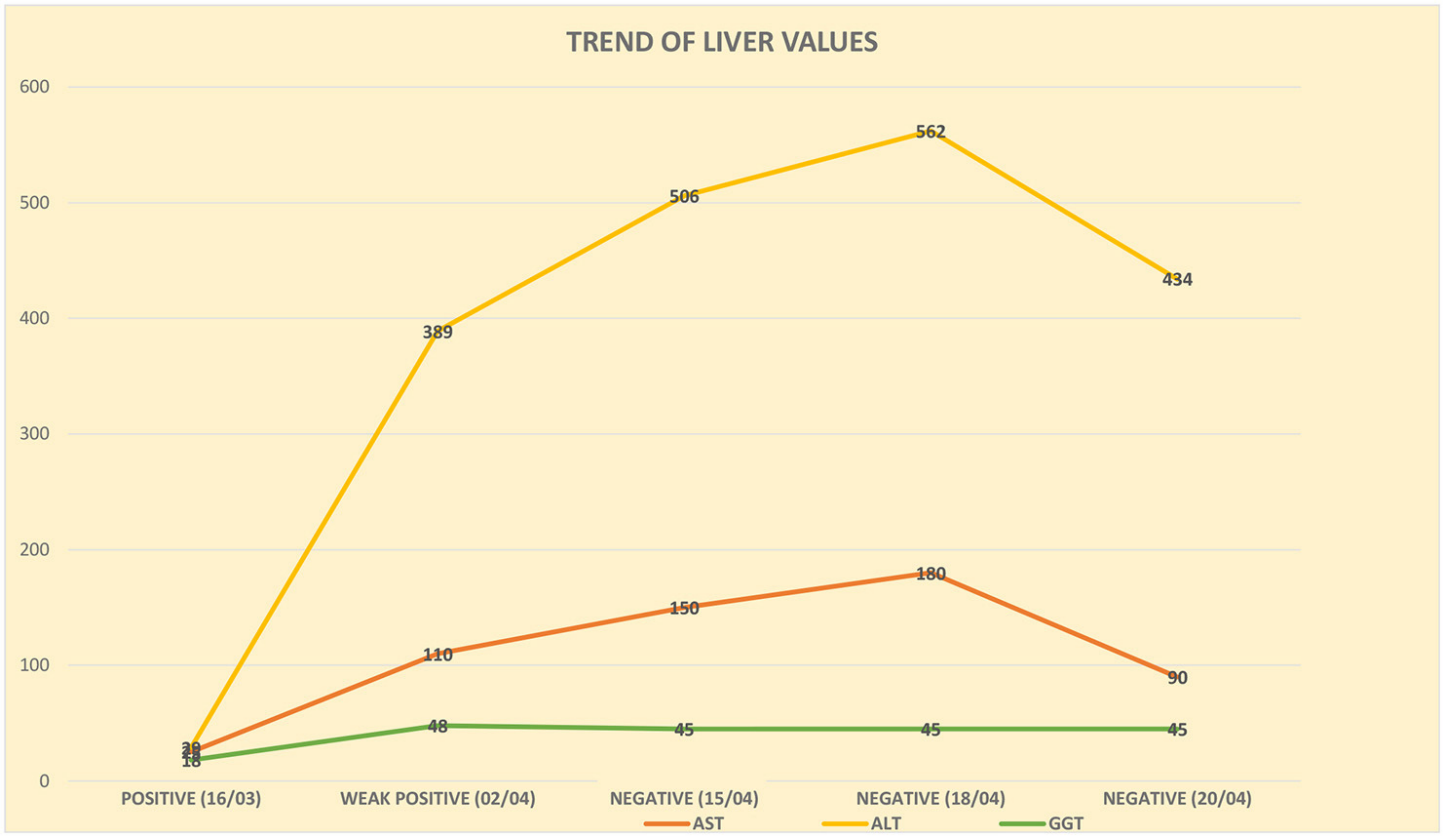
Trend of liver values.

In the first phase of hospitalization, we prescribed the ongoing psychopharmacological therapy with lithium carbonate (600 mg daily) and benzodiazepines and we started the administration of slow titration SSRI (sertraline). Monitoring of blood chemistry tests showed a progressive increase of AST, ALT e GGT (Figure 1) compared to the beginning when the nasopharyngeal swab tested positive for SARS-CoV-2.

On the 2nd day of hospitalization, a consultation was carried out with the infectious disease specialist who suggested to introduce an experimental therapy based on hydroxychloroquine with an initial loading dose of 800 mg/day for the first day, and then 400 mg/day on the following days, for a total of 10 days.

Due to a significant increase in AST (97 U/L) and ALT (362 U/L) levels along with a moderately increased gamma-GT values (66 U/L) further laboratory investigations were needed to search for the antigens of the main hepatotropic viruses that were absent. Hydroxychloroquine therapy was suspended on 2/4/2020, one week after it was started.

Furthermore, the execution of an abdominal ultrasound did not show morphological and structural alterations of the liver and biliary tract; finally, a parasitological examination was carried out on the stool, which was also negative on three samples. The consultation with the hepatologist confirmed the presence of drug-induced liver disease rather than a direct damage from SARS-CoV-2.

There was a weight loss of about 7 kg in 2 weeks with a change in the BMI from 24.22 to 21.8. The EDI-2 (10) was administered (Table 2) which revealed a significantly altered score in the Inadequacy (19) and Maturity fears (19) subscales.

**Table 2:** Eating disorder inventory.

**EDI-2 subscales**	
Ascetism	7
Body dissatisfaction	9
Bulimia	0
Drive for thinness	14^*^
Interpersonal distrust	16^*^
Impulse regulation	16^*^
Ineffectiveness	19^*^
Interceptive awareness	6
Maturity fears	19^*^
Perfectionism	0
Social insecurity	13^*^

“*” *refers to the EDI subscales in which the patient scored significantly*.

We observed a more rapid remission of the psychopathological picture as compared to the gastrointestinal picture and to the general clinical conditions, with regression of self-injurious thoughts and a stabilization of the dysthymia toward euthymia.

Two weeks after the diagnosis of SARS-CoV-2 infection, the nasopharyngeal test was repeated and was still weakly positive; subsequently, the test was repeated after a week and proved negative, and this result was confirmed by another molecular test on nasopharyngeal swab at 24 h. With the resolution of the infection, there has been a progressive improvement of gastro-intestinal symptoms with the patient gradually starting to ingest food. Despite this, the liver function indices remained altered even at the end of the hospitalization, 35 days after the entry/admission and after the positivity to SARS-CoV-2 was detected, with ALT values equal to 434 U/L and AST values equal to 90 U/L, so much so that an outpatient hepatological follow-up at our hospital was required.

## Discussion

In the first place this case history raises questions regarding differential diagnosis. The overlapping of psychopathological symptoms with the gastrointestinal manifestations of COVID-19, subject to ongoing examination, have made both diagnosis and treatment complex. Administering the EDI-2 consisting of 91 questions divided into 11 subscales, enabled us to exclude a concomitant eating disorder in the patient, despite her medical history showing a predisposition, above all in new acute phases of depression, for restrictive food intake and self-induced vomiting. The subscales in which she scored significantly were those of “drive for thinness”, “interpersonal distrust”, “impulse regulation”, “social insecurity”, “maturity fears” and “inadequacy”. The patient scored highest (19) on the latter two subscales. Total scoring may be related to the personological pattern associated with the patient's Borderline Personality Disorder, marked by poor self-image, feelings of uselessness, emptiness, inadequacy and lack of control over relational dynamics, associated with a tendency to regress and shelter behind childish behaviors due to a fear of maturity. Moreover, these characteristics correspond to clinical elements already highlighted during previous hospital stays.

As further evidence of the correlation of gastrointestinal symptoms with a SARS-CoV-2 infection we note that psychopathological symptoms improved rapidly as opposed to the patient's overall condition. The patient manifested a regression of self-harm ideation and a clear improvement of her dysthymia prior to the remission of nausea, vomiting and diarrhea, to the point that her hospital stay was extended to 4 weeks in order to cure the infection. When the patient entered the ward we were also able to ascertain the presence of a depressive episode by means of the Hamilton D evaluation scale. We chose HRSD supposing that physical symptoms were linked with depressive episode as previously observed.

The patient was put on a course of slow titration SSRI antidepressants so as not to worsen her gastrointestinal symptoms.

Implementing hospital protocol for cases testing positive for Sar-Cov2 enabled a close monitoring of blood chemistry tests considered to be useful in controlling the development of COVID-19. The discovery of significantly altered liver function made the treatment of the infection critical, requiring greater caution with regard to the titration of the antidepressant drugs prescribed.

One problem we faced was discriminating between potential liver damage caused by SARS-CoV-2 and iatrogenic damage caused by hydroxychloroquine used experimentally to modulate the inflammatory response to the infection, or both mechanisms. The persistence of gastrointestinal symptoms, already evidenced from the second day of hospitalization on our ward, despite treatment with hydroxychloroquine, led us to suspend this treatment after a week on the advice of the infectious diseases specialist. Subsequently, we witnessed a clinical improvement with the patient gradually starting to ingest food and a cessation of nausea and vomiting after meals. Two days after this improvement a nasopharyngeal swab proved negative. Following this result we were inclined to see a close correlation between viral load negativization and the improved clinical picture.

The infectious diseases specialist speculated that treatment with hydroxychloroquine caused the iatrogenic damage previously documented in the literature and that the same treatment used as preventive medicine for COVID-19 was of dubious efficiency (11). Nowadays the use of hydroxychloroquine in COVID-19 is not recommended (12).

In our case study other specialists excluded the direct liver damage by SARS-CoV-2 which is not confirmed even by recent studies that recommend to better investigate the causes of liver injury in patients with COVID-19 and the effect of treatment for COVID-19 on the liver (13).

We had a constant dialogue with the patient and her family. Management of side effects complicated hospital admission, prolonged the hospital stay and determined careful handling of the patient relationship and relations with family members.

## Conclusions

The current health emergency has severely impacted the Italian National Health Service and necessitated a reorganization of Mental Health Services and Facilities in order to meet social distancing requirements and to curb the spread of the epidemic. In our case it has proved necessary to set up a SPCD COVID ward dedicated to accommodating patients with acute disorders who have been tested molecularly.

It is well-known that access to general health services on the part of psychiatric patients is restricted and that often symptoms manifested by patients are underrated and attributed to their history of psychiatric disorders. In our case too, the patient visited Accident and Emergency twice in the days leading up to her admission manifesting a fever which was noted in her medical history. The patient only underwent a diagnostic test for SARS-CoV-2 upon entry to the SPDC. These circumstances led us to urge Emergency Services to carry out a more attentive evaluation of psychiatric patients as a piece of an overall picture taking into account general health and correlated COVID-19 symptoms in a population of fragile and complex patients who are also at greater risk due to a high frequency of unpredictable and promiscuous behaviors.

This case history refocuses attention on the importance of psychiatric medical history, the correct use of evaluation scales complementary to a diagnosis, especially where it is difficult to establish a differential diagnosis, prescription of treatment and management of complications. In this case report we suggest to test the patient with specific scales in order to make a differential diagnosis between gastrointestinal COVID-19 and eating disorder.

During this pandemic, specialists from various fields were called upon to support teams working on wards reserved for COVID-19 patients. There was a clear need to acquire new skills and to provide a clinical multidisciplinary evaluation by administering both psychological and laboratory tests.

We were in the first critical phase of the pandemic and at that time it has been very difficult to deal with patients with undergoing infection and the presentation symptoms widely recognized were respiratory. Gastrointestinal symptoms and hepatitis were still being studied.

The main limitation of this paper is the only one case report in our experience in the first pandemic period. It's important to add other similar cases to improve our skills to recognize covid symptoms between psychiatric symptoms and drug side effects (i.e., Hydroxychloroquine).

## Data Availability Statement

The original contributions presented in the study are included in the article/supplementary material, further inquiries can be directed to the corresponding author/s.

## Author Contributions

All authors listed have made a substantial, direct, and intellectual contribution to the work and approved it for publication.

## Conflict of Interest

The authors declare that the research was conducted in the absence of any commercial or financial relationships that could be construed as a potential conflict of interest.

## Publisher's Note

All claims expressed in this article are solely those of the authors and do not necessarily represent those of their affiliated organizations, or those of the publisher, the editors and the reviewers. Any product that may be evaluated in this article, or claim that may be made by its manufacturer, is not guaranteed or endorsed by the publisher.

## Footnotes

^1^ https://www.epicentro.iss.it/coronavirus/aggiornamenti

^2^ https://www.who.int/news-room/commentaries/detail/modes-of-transmission-of-virus-causing-covid-19-implications-for-ipc-precaution-recommendations
